# Antioxidant Properties of Essential Oil Extracted from *Pinus morrisonicola* Hay Needles by Supercritical Fluid and Identification of Possible Active Compounds by GC/MS

**DOI:** 10.3390/molecules201019051

**Published:** 2015-10-20

**Authors:** Ming-Ching Cheng, Wen-Hua Chang, Chih-Wei Chen, Wen-Wing Li, Chin-Yin Tseng, Tuzz-Ying Song

**Affiliations:** 1 Department of Health Food, Chung Chou University of Science and Technology, Changhua 51591, Taiwan; E-Mails: m25522@yahoo.com.tw (M.-C.C.); transwen@dragon.ccut.edu.tw (W.-H.C.); ccwlly@dragon.ccut.edu.tw (C.-W.C.); wf09060225@yahoo.com.tw (W.-W.L.); president@dragon.ccut.edu.tw (C.-Y.T.); 2 Department of Bioindustry Technology, Dayeh University, Changhua 51591, Taiwan

**Keywords:** *Pinus morrisonicola* Hay, supercritical fluid extract, essential oil, lipid peroxidation, foam cell formation

## Abstract

Pine (*Pinus morrisonicola* Hay, PM) needles have been used as folk medicine for their antihypertension and lipid-lowering effects. As supercritical fluid extraction (SFE) is considered an ideal technique for the extraction of essential oil from plant materials, the present work investigated the optimal SFE conditions and the protective effects of different resulting fractions of PM needles on lipid peroxidation and foam cell production in macrophages. Nine PM needle extracts (PME1–9) were obtained in 1%–4% yields using different SFE conditions, of which PME1 had the lowest yield (1.1%) and PME3 the highest (3.9%). PME3 exhibited lower cytotoxic effects and stronger inhibition of lipid peroxidation and formation of foam cell in RAW 264.7 macrophages than those of other PME extracts. PME3-1 purified from PME3 by column and thin layer chromatography inhibited LDL oxidation more effectively than did PME3 in a cell-free system oxidized by Cu^2+^. PME3-1 dose-dependently (25–100 μg/mL) decreased conjugated diene levels and foam cell formation induced by ox-LDL. GC/MS analyses revealed that 1-docosene, neophytadiene, and methyl abietate were increased 5.2-, 1.7- and 4.3-fold in PME3-1 relative to PME3. A new hydrocarbon compound, cedrane-8,13-diol, was identified in PME3-1. Overall, the present study demonstrates the optimal extraction conditions of SFE of PM and identifies the most potent antioxidant fractions and possible active compounds in PM.

## 1. Introduction

Oxidation of low-density lipoprotein (LDL) is crucial in plaque formation and the onset of atherosclerosis. The LDL is subject to oxidative modification to form oxidized LDL (ox-LDL) due to its high content of unsaturated fatty acyl groups [1]. Ox-LDL can cause macrophage growth inhibition, induction of monocyte differentiation into macrophages, promotion of its uptake by macrophages through scavenger receptors, and cytotoxicity of macrophages [2]. Consequently, macrophage apoptosis plays an important role in the development of atherosclerotic lesion. Occurrence of macrophage apoptosis in human atheroma has been demonstrated [3,4]. Much evidence has implicated that atherosclerosis has a close relationship with ox-LDL, which is clearly a main risk factor for coronary heart disease. In contrast, much evidence shows that dietary antioxidants (e.g., polyphenol and flavonoid) can potentially protect against LDL oxidation [5]. Epidemiological studies have repeatedly shown that diets rich in fruit and vegetables containing antioxidants are well associated with lower risks of cardiovascular diseases [6,7]. Thus, increased intakes of dietary antioxidants may be useful to prevent LDL oxidation and the atherosclerotic progression.

Pine (*Pinus morrisonicola* Hay, PM), also called Taiwanese short-leaf pine, belongs to the Pinaceae family [8,9]. PM needles have been used as tonic drinks and a folk medicine for antihypertension in Asia [8]. PM needles extracted with various solvents (water, ethanol, ethyl acetate) exhibit *in vitro* biological functions, including antioxidant activity, anti-mutagenicity, anti-inflammatory actions, inhibition of the growth of human leukemic cell line U937, and antitumor effects [9,10,11,12,13]. In addition, the ethyl acetate extract of PM needle was shown to inhibit Cu^2+^-induced LDL oxidation and attenuate excessive NO generation in mouse RAW 264.7 macrophages treated with lipopolysaccharide (LPS) [14].

The solvent lipid extraction methods for plant essential oils are being superseded by supercritical fluid extraction (SFE) methods, as the latter are rapid, automatable, selective, and can avoid the use of large amounts of solvent [15]. Among different supercritical fluids, CO_2_ is considered environmentally friendly, safe, non-toxic, non-carcinogenic, non-flammable, inexpensive, and to have modest critical conditions. The selectivity of the supercritical CO_2_ (SF-CO_2_) can be adjusted by varying temperature and pressure to obtain fractions consisting of desirable compounds [16]. It has been shown that the essential oils can be obtained from plants by SF-CO_2_ extraction (SFE-CO_2_) methods [17] and that the essential oil has hypolipidemic effects *in vivo* [18] and can potentially be used as a natural antioxidant and antimicrobial agent in food processing [19,20]. Besides, the majority of the essential oils are classified as Generally Recognized As Safe (GRAS) ingredients [21]. These features of SFE are ideal for extraction of essential oil from plant materials. However, to our knowledge, no reports have been available on the use of SFE for PM needle essential oil. Therefore, the present study aimed to determine the optimal conditions of SFE for PM needles and whether the PM extracts (PME) exert antioxidant activities in RAW 264.7 macrophages treated with ox-LDL. Furthermore, we analyzed the possible bioactive compounds in PME using GC/MS.

## 2. Results

### 2.1. Effect of PME on Cell Viability of Macrophages

**Figure 1:**
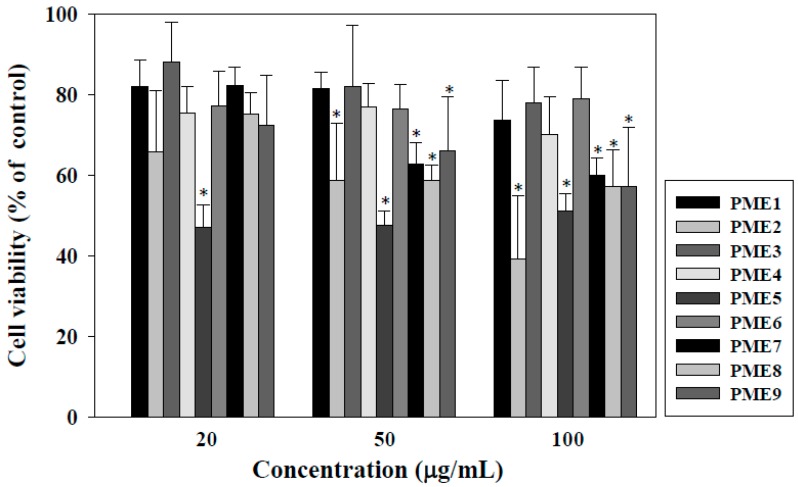
Effects of *Pinus morrisonicola* extract (PME) fractions 1 to 9 (PME1–9) on the viability of RAW 264.7 macrophages for 24 h. * indicate significantly different by comparison with the control (*p* < 0.05).

The viability of macrophages was determined to select the non-cytotoxic concentrations of PME for the following experiments. As shown in Figure 1, PME2 and PME5 displayed strong cytotoxic effects by inhibiting cell viability of 40% and 50% at 100 μg/mL, respectively, whereas PME fractions 1, 3, 4, and 6 exhibited relatively low cytotoxic activities; each fraction retained about 80% cell viability at the concentration of 100 μg/mL. Thus, we selected PME 1, 3, 4 and 6 for the following antioxidant tests.

### 2.2. Effect of PME on Lipid Peroxidation in Macrophages Treated with ox-LDL

**Figure 2:**
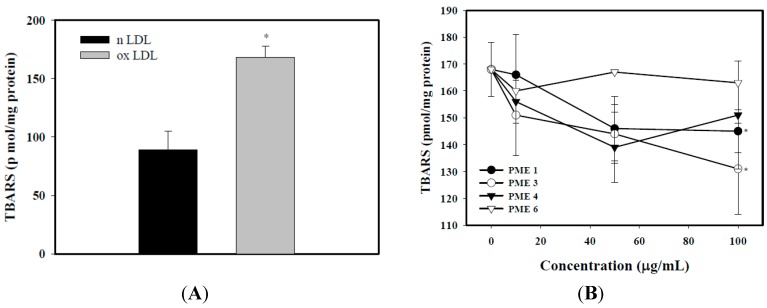
Thiobarbituric acid-reactive substances (TBARS) in RAW 264.7 macrophages incubated with n-LDL or ox-LDL (100 μg/mL). (**A**) * indicate significantly different by comparison with n-LDL (*p* < 0.05); (**B**) The inhibitory effects of *Pinus morrisonicola* extracts (PME) fractions 1, 3, 4, and 6. Asterisks are significantly different (*p* < 0.05) as compared to zero concentration of PME. Data are expressed as means ± SD of three separate experiments.

As shown in Figure 2A, levels of TBARS in macrophages treated with ox-LDL were significantly increased, as compared to those treated with n-LDL (*p* < 0.05). The inhibition of lipid peroxidation (levels of TBARS) in macrophage by PME 1, 3, 4, and 6 was shown in Figure 2B. The results reveal that PME 1 and 3 significantly inhibited the levels of TBARS, with an inhibition of 29.1% and 46.8%, respectively, at the concentration of 100 μg/mL (*p* < 0.05). However, the other PME extracts did not significantly inhibit lipid peroxidation in macrophages treated with Cu^2+^ (*p* > 0.05). As a result, we used PME3 for the following experiments.

### 2.3. Effect of PME3 on Foam cell Formation in Macrophages Treated with ox-LDL

**Figure 3:**
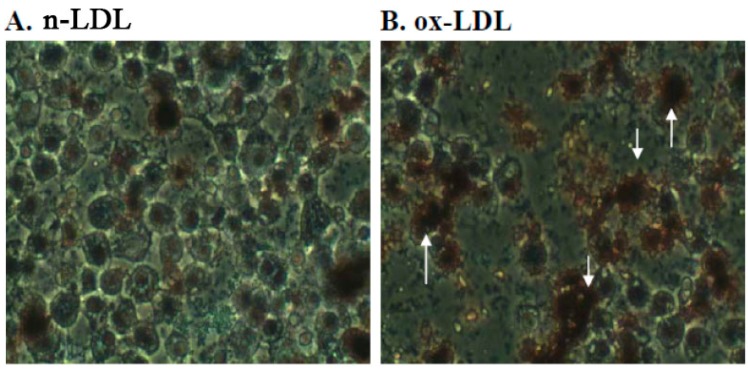

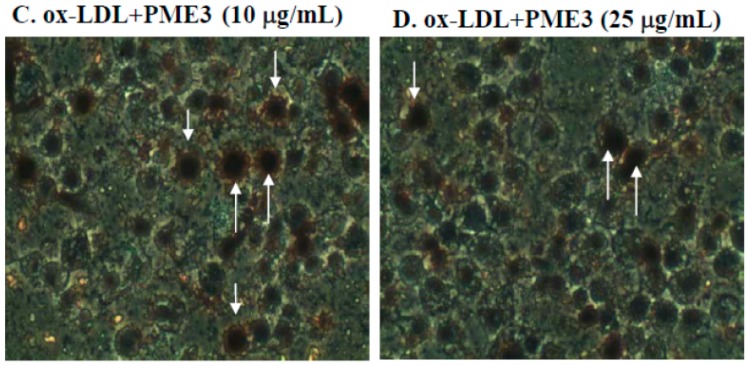
Effects of *Pinus morrisonicola* extract fraction 3 (PME3) on foam cell formation in RAW 264.7 macrophages incubated with ox-LDL for 24 h. (**A**): RAW 264.7 cells treated with native LDL (n-LDL); (**B**): RAW 264.7 cells exposed to ox-LDL (ox-LDL); (**C**,**D**): RAW 264.7 cells exposed to ox-LDL in the presence of 10 μg/mL (**C**) and 25 μg/mL (**D**) of PME3. Arrow heads indicate foam cell formation.

Because PME3 RAW 264.7 macrophages treated with n-LDL for 24 h caused little or no formation of foam cell (Figure 3A). In contrast, RAW 264.7 macrophages treated with Cu^2+^-oxidized LDL for 24 h resulted in significantly increased formation of foam cells (Figure 3B), an indication of increased uptake of ox-LDL. However, when PME3 was included in the macrophages treated with LDL and CuSO_4_, the foam cell formation (*i.e.*, ox-LDL accumulation) was significantly reduced (Figure 3C,D). The effect of PME3 was stronger at the higher concentration (25 μg/mL, Figure 3D) than at the lower concentration (10 μg/mL), whereas PME fractions 1, 4, and 6 added at the concentration of 25 μg/mL did not display significant inhibitory effects (data not shown).

### 2.4. Fractionation of PME3 by Column Chromatography and Thin-Layer Chromatography

PME3-1, PME3-2, PME3-3 and PME3-4 were isolated from PME3 by silica gel column chromatography using an elution system of *n*-hexane–ethyl acetate = 1:0.2. The overall recovery of PME isolates from the column is 90%–95% at a loading capacity of 150 mg of PME per 10 g of silica gel. The complete separation of four PME isolates (PME3-1, PME3-2, PME3-3 and PME3-4) is also achieved by one-dimensional silica gel thin-layer chromatography with the solvent system *n*-hexane–ethyl acetate = 1:0.2. The elution sequence of PME3 was assigned as PME3-1 (tubes 1–6), PME3-2 (tubes 8–14), PME3-3 (tubes 20–32), and PME3-4 (tubes 251–270), and their yields were about 60%, 28%, 3%, and 0.5%, respectively, and their Rf values were 0.91, 0.50, 0.26, and 0.10, respectively (Table 1).

**Table 1:** Tube number, Rf value and yields of *Pinus morrisonicola* extract (PME) fractions 3-1 to 3-4 (PME3-1 to 3-4) by thin layer chromatography with the solvent system of *n*-hexane:ethyl acetate = 1:0.2.

PME3 Fractions	Tube Number	Rf Value	Yields (%)
PME3-1	1–6 *	0.91	60
PME3-2	8–14	0.50	28
PME3-3	20–32	0.26	3
PME3-4	251–270	0.10	0.5

*: The volume of the collecting tube is 10 mL.

### 2.5. Inhibitory Effects of PME3-1 on Conjugated Diene in Cell-Free n-LDL Oxidized by Cu^2+^

**Figure 4:**
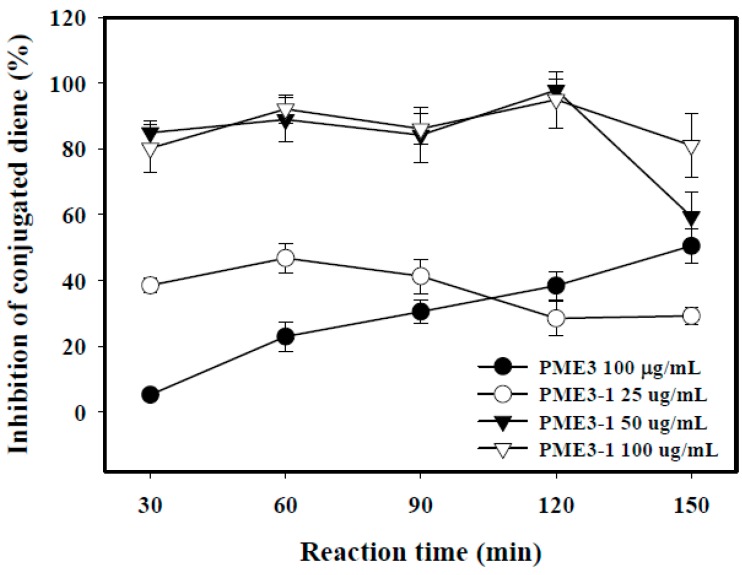
Inhibitory effects of the *Pinus morrisonicola* extract fraction 3 (PME3) and PME3-1 on conjugated diene levels in cell free n-LDL oxidized by Cu^2+^. Native LDL (n-LDL, 100 μg/mL) was pre-incubated with PME3 (100 μg/mL) or increasing concentration of PME3-1 (25–100 μg/mL) for 1 h. Conjugated diene was induced by the addition of CuSO_4_ (10 μM) at 37 °C for 150 min.

As shown in Figure 4, PME3-1 inhibited conjugated diene formation in cell-free n-LDL induced by ox-LDL in a dose-dependent manner (from 25–50 μg/mL). The maximum inhibition on conjugated diene of PME3-1 added at 50 and 100 μg/mL was between 80% and 100% at 120 min, after which time the effect weakened substantially. In contrast, PME3 itself had little inhibition at 30 min of incubation, but the inhibitory effect increased gradually with time and reached almost the same inhibition as was the PME3-1 added at 50 μg/mL at 150 min of incubation.

### 2.6. Inhibitory Effects of PME3-1 on Foam Cell Formation in Macrophages Treated with ox-LDL

**Figure 5:**
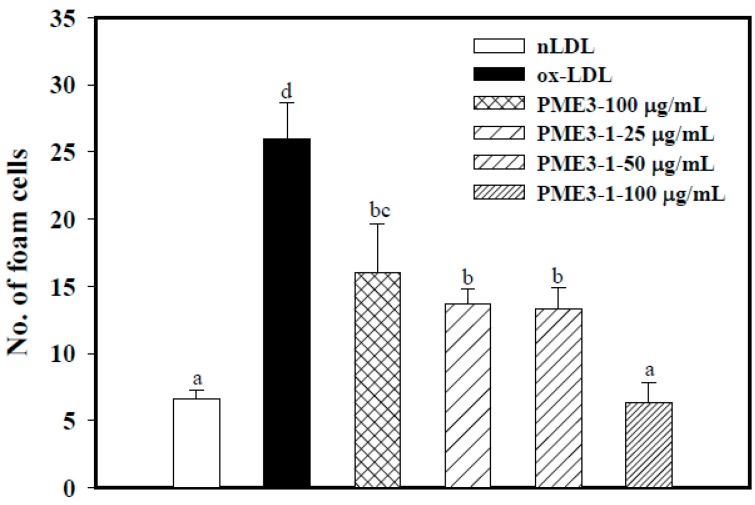
Inhibitory effects of PME3-1 on foam cell formation in RAW 264.7 macrophages induced by oxidized-LDL. ox-LDL was induced by the addition of CuSO_4_ (10 μM) to n-LDL (100 μg/mL) at 37 °C for 24 h. Then, ox-LDL (or n-LDL), PME3, or PME3-1 was co-incubated with macrophages for 24 h. Data are expressed as means ± SD of three separate experiments. Values not sharing an alphabetic letter are significantly different (*p* < 0.05).

PME3-1 strongly and concentration-dependently inhibited foam cell formation in macrophage induced by ox-LDL (Figure 5). Incubation of RAW 264.7 cells with ox-LDL for 24 h resulted in significant uptakes of ox-LDL, causing higher intracellular foam cell accumulation (25%), as compared to control cells that were exposed to LDL alone (n-LDL; 7%). Addition of PME3-1 extract to ox-LDL treated macrophages significantly reduced ox-LDL uptake and formation of foam cells in RAW 264.7 macrophages, and the foam cell formation was completely inhibited by PME3-1 at the concentration of 100 μg/mL (Figure 5).

### 2.7. GC/MS Analysis of PME3 and PME3-1

**Figure 6:**
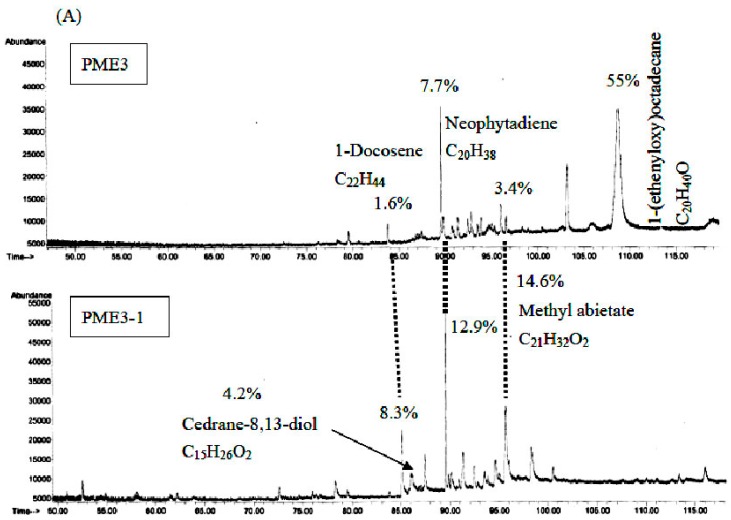

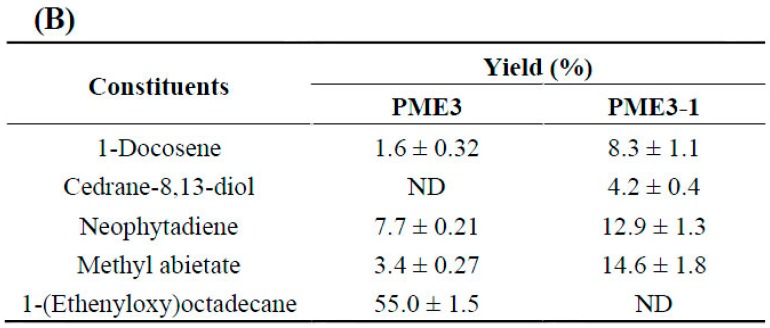
GC/MS analysis of hydrocarbon compounds in *Pinus morrisonicola* extract fraction 3 (PME3) and PME3-1. (**A**): GC/MS profile; (**B**): the constituent yield of PME3 and PME3-1. ND: not detectable.

The chemical components of the PME3 and PME3-1 were determined using GC/MS. Figure 6 shows that the hydrocarbon compounds such as 1-docosene, neophytadiene, and methyl abietate in PME3-1 increased 5.2-, 1.7-, and 4.3-fold, respectively, as compared to those in PME3. GC/MS analyses revealed a hydrocarbon compound cedrane-8,13-diol (4.2%) in PME3-1. Cedrane-8,13-diol has not been identified in PM previously. In the present study, this compound was found only in PME3-1 in sufficient amounts following the fractionation of PME3 by column chromatography and thin-layer chromatography.

## 3. Discussion

The aim of this study was to evaluate optimal extraction conditions of SFE for PM (*i.e.*, temperature, pressure, time, and adjuvant volume) on antioxidant activity and to identify the active fractions and compounds in PM. The SFE conditions for extracting essential oil from PM reported in the present study may be applicable to the extraction of essential oil or pigment in various plants. The results reveal that the optimal SF-CO_2_ conditions for the antioxidant activity of PM were 25 MPa for 15 min at 40 °C. Under these conditions, the yield of SF extracts from PM increased with increasing ethanol volume. As indicated by Lin *et al.* [22], the SF-CO_2_ system has low polarity at high pressure, so only traces of polar compounds were extracted and often only the low polarity compounds, such as alkanes, alkoxy, aliphatic aldehydes and fat, were obtained. Consequently, SF with carbon dioxide requires the addition of small amounts of more polar solvents (such as ethanol) or modifiers to increase the solvent strength of the mobile phase, deactivate active sites on the surface of the packing material, and allow the elution of polar compounds with high efficiency [23]. It has been shown that plant extracts that have higher polarity are less cytotoxic [24]. We found that the inhibitory effects of PME on cell viability decreased with increasing polarity (*i.e.*, no ethanol added) and that PME2 and PME5 had the strongest cell cytotoxicity (cell viability decreased to 40% and 50%, respectively, at the concentration of 100 μg/mL). However, PME fractions 1, 3, 4, and 6, which had higher polarity (75%–80%) displayed less cytotoxicity than the other PME fractions. In addition, the extracts obtained by higher extraction temperature had higher cytotoxic effects (*i.e.*, PME fractions 7, 8, and 9). These results indicate that the lower polarity or higher temperature in the extraction conditions of SF-CO_2_ results in higher cytotoxicity in PME fractions.

Among the several SFE fractions of PM needles, PME3 was not cytotoxic and was the only fraction that effectively inhibited lipid peroxidation in RAW 264.7 macrophages. We then analyzed the components in PME3 and found that PME3 contained polyphenols, flavonoids, and *p*-coumaric acid (14,500, 960, and 460 μg/g) (data not shown). Yen *et al.* [14] indicated that the ethyl acetate extracts of PM may contribute to its anti-atherosclerotic effects by inhibition of LDL oxidation and that the main bioactive compounds in the ethyl acetate extracts of PM are epicatechin and *p*-coumaric acid. Fang *et al.* [25] also indicated that the acetone extract of heartwood from PM contains various flavonoids and stilbenes.

We further fractionated PME3 by column and thin-layer chromatography to obtain PME3-1 through PME3-4, and we found that the inhibitory effect of PME3-1 on conjugated diene and foam cell formation was about two times higher than those of PME3. Furthermore, we analyzed the components in PME3-1 by GC/MS, and our data suggest that the essential oil compounds, such as 1-docosene, neophytadiene, and methyl abietate, could be the major active components in PME3-1. This study represents the first to explore the appropriate condition to obtain SFE-CO_2_ extracts from PM and to identify the possible active compounds responsible for their antioxidant activities.

Regarding the active compounds in PM needle essential oil, a new abietic acid-type diterpene glucoside and terpinolene were found to exhibit remarkable protection against LDL-oxidation [26,27]. In addition, 3-methylene-7,11,15-trimethylhexadec-1-ene (neophytadiene) was found in the hexane extracts of *Bursera simaruba* (L.) Sarg. leaves which displays anti-inflammatory activities on carrageenan-induced inflammation in rats, and the effect observed at the dose of 1.50 g/kg·bw was comparable to that of phenylbutazone at the dose of 80 mg/kg·bw [28]. The same study also identified a hydrocarbon compound, cedrane-8,13-diol, a kind of sesquiterpenoid, which exhibits hypolipidemic and antioxidant activities [18]. In the present study, four major constituents were identified in PM needle essential oil by GC/MS in PME3-1, namely 1-docosene, cedrane-8,13-diol, neophytadiene, and methyl abietate, and their contents were 5.2-, 4.2-, 1.7- and 4.3-fold, respectively, higher than those of PME3. However, 1-(ethenyloxy)octadecane detected in PME3 was not found in PME3-1. Though we did not prove that cedrane-8, 13-diol, neophytadiene and methyl abietate were the active components in PME3-1. Evidence shows that cedrane-8,13-diol, neophytadiene and methyl abietate have hypolipidemic and antioxidant activities [18,26,27,28]. Thus, we speculate that cedrane-8,13-diol, neophytadiene and methyl abietate may participate in the antioxidant activity of PM3-1.

In conclusion, the present results demonstrate that the optimal operation conditions of SFE-CO_2_ for antioxidant activity of PM are 25 MPa for 15 min at 40 °C. The PME3 fraction of the PM needle is able to inhibit lipid peroxidation and foam cell formation in RAW 264.7 cells. We further showed that PME3-1 may contribute to the biological function of PME3 and that cedrane-8,13-diol may be the major active compound in PME3. Further studies are needed to ascertain the hypolipidemic effect of PME3-1 *in vivo* and the major active compounds in PME3.

## 4. Experimental Section

### 4.1. Chemical and Reagents

Sodium nitrite, albumin, bovine, potassium chloride, sodium bicarbonate, lipopolysaccharide (LPS), Oil Red O (ORO), 3-(4,5-dimethylthiazol-2-yl)-2,5-diphenyltetrazolium bromide (MTT) were purchased from Sigma Co. Ltd. (St Louis, MO, USA). Copper sulfate pentahydrate, sodium chloride, trichloroacetic acid, copper sulfate pentahydrate, and dimethyl sulfoxide (DMSO) were obtained from Merck (Darmstadt, Germany). Pine (*P. morrisonicola* Hay) needles were kindly supplied by the KARA Legend Biotechnology Co., Ltd. (Pingchen, Taoyuan County, Taiwan). All other chemicals were reagent grade.

### 4.2. Sample Preparation

Fresh pine (*P. morrisonicola*) needles were freeze-dried, ground to powder, passed through a 100 mesh sieve and stored at −20 °C until use.

### 4.3. Supercritical Fluid Extraction

The model 260D system for supercritical fluid extraction was purchased from Teledyne ISCO (Lincoln, NE, USA). Twenty grams of powdered sample of pine needles were placed in the 30-mL extraction vessel and set at 40, 50, 60 °C. The pressure was adjusted at 15, 20, and 25 MPa, respectively. Ethanol was used as an adjuvant solvent, and the volume was 1, 2, and 3 mL, respectively. The flow rates for CO_2_ were fixed at 6 mL/min. Once the set temperature and pressure were achieved after turning on the injection valve and the system was in equilibrium, the extraction was carried out for 15 min in each experimental run. The final extract was collected in a flask connected to the back pressure regulator. The solvent was evaporated by drying under vacuum using rotary evaporator; the extract was weighed to obtain the yield (calculated as percentage of 20-g pine needles converted into extract by SF) and stored at −20 °C before further analysis of bioactive compounds. Conditions and of the yields of supercritical fluid extraction for PME are shown in Table 2. Fractions of PME1 to 9 were obtained by different supercritical extraction conditions with 1%–4% yields, of which PME1 had the lowest yield (1.1%) and PME3 had the highest (3.9%).

**Table 2:** Conditions and the yields of supercritical fluid extraction for *Pinus morrisonicola* extract (PME).

Extract	Temperature (°C)	Pressure (MPa)	Time (min)	Adjuvant (mL)	Yield (%)
PME 1	40	15	5	1	1.1 ± 0.04 *
PME 2	40	20	10	2	3.2 ± 0.52
PME 3	40	25	15	3	3.9 ± 1.07
PME 4	50	15	20	3	3.7 ± 0.90
PME 5	50	20	25	1	3.4 ± 0.02
PME 6	50	25	15	2	2.7 ± 0.27
PME 7	60	15	25	2	2.8 ± 0.50
PME 8	60	20	15	3	3.7 ± 0.05
PME 9	60	25	20	1	2.5 ± 0.06

*: Data are expressed as means ± SD of three separate experiments.

### 4.4. Separation and Characterization of PME3

Fractions of PME3 are readily separated by silica gel column chromatography using an elution system of *n*-hexane–ethyl acetate = 1:0.2 (200 mL/bottle). The overall recovery of PME3 isolates from the column is 90%–95% at a loading capacity of 150 mg of PME per 10 g of silica gel. The complete separation of four PME3 subfractions (PME3-1, PME3-2, PME3-3 and PME3-4) was also achieved by one-dimensional silica gel thin-layer chromatography with the solvent system *n*-hexane–ethyl acetate = 1:0.2.

### 4.5. GC/MS Analysis of Chemical Components in PME3 and PME3-1

A HP6890 series GC coupled with a 5973 Network Mass Selective Detector (Agilent Technologies, Santa Clara, CA, USA) was used for quantification and determination of the chemical components in the PME3 and PME3-1 of the pine extract. The SF-CO_2_ extraction for PM in our study primarily fractionated the compounds with low polarity and low boiling point. To some extent, increasing the initial temperature of column will increase the polarity or boiling point of compounds [18]. Thus, we used low temperature and a high retention time to identification of the compounds. A capillary column type DB-1 (i.d. 0.25 mm × 60 m; membrane thickness, 0.25 μm) was used. Helium was used as the carrier gas and operated at a flow rate of 1 mL/min. The ionization potential used was 70 eV. The temperature of the ion source was set at 230 °C. The flux ratio was set at 50:1. Initially, the temperature was set at 40 °C for 10 min, then programmed at 1 °C/min up to 150 °C, than programmed at 5 °C/min up to 200 °C, then programmed at 11 °C/min up to 250 °C, and held at this temperature for 10 min. Quantification of PME3 and PME3-1. Aliquots (1.0 μL) of the extracts were, respectively, measured with a GC microsyringe from fractionates of pine extract and analyzed with GC/MS. Quantification of each constituent in PME3 and PME3-1 were calculated from the integrated diagrams obtained by Equation (1):

Q = A × Y
(1)

where Q = the quantity of each volatile constituent in extracts PME3 and PME3-1, A = the percent peak area in the gas chromatograms occupied by each constituent, and Y = the recovery yield of extracts.

The molecular weight and chemical structure splitting pattern of each peak in the GC chromatogram of PME3 and PME3-1 were compared with known compounds in the databases such as the Wiley MS Chemstation Libraries, NBS Computer Data [29], and reference mixture of *n*-alkanes (C5–C25) to calculate the retention indices (RI) from the retention time for each component. A compound is identified, when the similarity of the peak with the library compound is above 90%.

### 4.6. Lipoprotein Separation

Human plasma was obtained from the Taichung Blood Bank (Taichung, Taiwan), and LDL was isolated using sequential ultracentrifugation (*p* = 1.019–1.063 g/mL) in KBr solution containing 30 mM EDTA, stored at 4 °C in a sterile, dark environment, and used within 3 days as previously described [30]. Immediately before the oxidation tests, LDL was separated from EDTA and from diffusible low molecular mass compounds by gel filtration on PD-10 Sephadex G-25 M gel (Amersham Pharmacia Biotech Inc., Piscataway, NJ, USA) in 0.01 mol/L phosphate-buffered saline (136.9 mmol/L NaCl, 2.68 mmol/L KCl, LDL (100 μg of protein/mL) to obtain the native-LDL (n-LDL) for various tests in a cell-free LDL oxidation system and in macrophages.

### 4.7. Cell Culture and Treatment with Oxidized LDL

RAW 264.7 cells (BCRC 60001), the murine macrophage cell line, were obtained from the Bioresources Collection and Research Center, Food Industry Research and Development Institute (Hsinchu, Taiwan). Cells were cultured in endotoxin-free Dulbecco’s modified Eagle’s medium, supplemented with 10% fetal bovine serum and penicillin (100 units/mL) plus streptomycin (100 mg/mL) and maintained at 37 °C in 5% CO_2_. To induce lipid peroxidation and foam cell formation, RAW 264.7 macrophages were treated with oxidized-LDL (ox-LDL) which was obtained by incubating n-LDL with CuSO_4_ (50 μM) at 37 °C for 24 h. To determine the protective effects of PME extracts, the macrophages were pretreated with PME extracts for 2 h before incubation with ox-LDL or n-LDL.

### 4.8. Determination of Cell Viability

A portion (0–100 μg/mL) of PME fractions 1 to 9 was incubated with RAW 264.7 macrophages at 37 °C for 24 h. Cell viability was measured using the 3-(4,5-dimethylthiazol-2-yl)-2,5-diphenyl tetrazolium bromide (MTT) assay according to the method of Denizot *et al.* [31]. Briefly, cells (1 × 10^4^) were dispensed into 96-well plates and sample (PME1–9) was added then incubation at 37 °C in humidified atmosphere containing 5% CO_2_ for 24 h. Then, culture solutions were removed and replaced by 90 μL culture medium. Ten microliters of sterile filtered MTT solution (5 mg/mL) in PBS was added to each well, reaching a final concentration of 0.5 mg MTT/mL. Un-reacted dye was removed after 5 h. The insoluble formazan crystals were dissolved in 200 mL/well dimethyl sulfoxide (DMSO) and measured spectrophotometrically at 570 nm. The cell viability was expressed as the percentage of cell growth compared to control non-treated cells (at which growth is considered 100%) and it was calculated by A570 nm (sample)/A570 nm (control) × 100.

### 4.9. Determination of Thiobarbituric Acid-Reactive Substances (TBARS) in Macrophages Treated with Oxidized LDL

Lipid peroxidation in RAW 264.7 macrophages was determined as TBARS using the TBA assay [32]. Briefly, a portion (0–100 μg/mL) of PME 1, 3, 4, and 6 was incubated with macrophages for 2 h before incubation with ox-LDL (100 μg/mL) at 37 °C for 24 h. Butylated hydroxytoluene (10 μL, 50 mM) was added to terminate peroxidation reaction, and the mixture was then added with 1 mL of 7.5% (*w*/*v*) cold trichloroacetic acid. After centrifugation, the supernatant was allowed to react with 1 mL of 0.8% (*w*/*v*) TBA in a boiling water bath for 45 min. After cooling, levels of TBARS were determined spectrofluorimetrically (at 515 nm excitation and 555 nm emission) using 1,1,3,3-tetraethoxypropane as the standard. Results are expressed as pmoles of TBARS content per milligram protein.

### 4.10. Determination of Foam cell Formation in Macrophages Treated with Ox-LDL

The foam cell formation assay was carried out by the method of Halvorsen *et al.* [33]. RAW 264.7 cells were incubated with ox-LDL (100 μg/mL) or native LDL (n-LDL) in the absence or presence of PME3 and PME3-1 (25–100 μg/mL) for 24 h. After incubation, the cells were washed once in ice-cold PBS (phosphate buffered saline, pH 7.4), followed by formaldehyde fixation for 30 min at room temperature (25 °C). Neutral lipids were stained using 0.5% oil-red-O in isopropanol for 60 min. The ox-LDL uptake was using microscopy.

### 4.11. Determination of Conjugated Diene Formation in Cell-Free LDL Oxidized by Cu^2+^

Native LDL (n-LDL, 100 μg/mL) was pre-incubated with PME1, 3, 4, and 6 (100 μg/mL) at room temp for 1 h followed by addition of CuSO_4_ (50 μM) at 37 °C for 150 min. Conjugated diene formation was monitored by the method described by Yen *et al.* [14]. The UV spectrum (OD_234nm_) was recorded, and the increase in conjugated diene expressed in relative unit was obtained from the absorbance at 234 nm.

### 4.12. Statistical Analysis

All statistical analyses were performed using SPSS for Windows, version 17 (SPSS, Inc., Chicago, IL, USA). Data are expressed as means ± SD and analyzed using one-way ANOVA followed by Duncan’s multiple range test. *p* < 0.05 is considered statistically significant.
